# Extremely anisotropic van der Waals thermal conductors

**DOI:** 10.1038/s41586-021-03867-8

**Published:** 2021-09-29

**Authors:** Shi En Kim, Fauzia Mujid, Akash Rai, Fredrik Eriksson, Joonki Suh, Preeti Poddar, Ariana Ray, Chibeom Park, Erik Fransson, Yu Zhong, David A. Muller, Paul Erhart, David G. Cahill, Jiwoong Park

**Affiliations:** 1 Pritzker School of Molecular Engineering, University of Chicago, Chicago, IL USA; 2 Department of Chemistry, University of Chicago, Chicago, IL USA; 3 Department of Materials Science and Engineering and Materials Research Laboratory, University of Illinois at Urbana-Champaign, Urbana, IL USA; 4 Department of Physics, Chalmers University of Technology, Gothenburg, Sweden; 5 James Franck Institute, University of Chicago, Chicago, IL USA; 6 School of Applied and Engineering Physics, Cornell University, Ithaca, NY USA

**Keywords:** Two-dimensional materials, Two-dimensional materials

## Abstract

The densification of integrated circuits requires thermal management strategies and high thermal conductivity materials^1–3^. Recent innovations include the development of materials with thermal conduction anisotropy, which can remove hotspots along the fast-axis direction and provide thermal insulation along the slow axis^4,5^. However, most artificially engineered thermal conductors have anisotropy ratios much smaller than those seen in naturally anisotropic materials. Here we report extremely anisotropic thermal conductors based on large-area van der Waals thin films with random interlayer rotations, which produce a room-temperature thermal anisotropy ratio close to 900 in MoS_2_, one of the highest ever reported. This is enabled by the interlayer rotations that impede the through-plane thermal transport, while the long-range intralayer crystallinity maintains high in-plane thermal conductivity. We measure ultralow thermal conductivities in the through-plane direction for MoS_2_ (57 ± 3 mW m^−1^ K^−1^) and WS_2_ (41 ± 3 mW m^−1^ K^−1^) films, and we quantitatively explain these values using molecular dynamics simulations that reveal one-dimensional glass-like thermal transport. Conversely, the in-plane thermal conductivity in these MoS_2_ films is close to the single-crystal value. Covering nanofabricated gold electrodes with our anisotropic films prevents overheating of the electrodes and blocks heat from reaching the device surface. Our work establishes interlayer rotation in crystalline layered materials as a new degree of freedom for engineering-directed heat transport in solid-state systems.

## Abstract (web summary)

Extremely anisotropic thermal conductors based on large-area van der Waals thin films with random interlayer rotations are reported here.

## Main

Anisotropic thermal conductors, in which heat flows faster in one direction than in another, can be characterized by the thermal conductivity anisotropy ratio *ρ* (= *κ*_f_/*κ*_s_) between the thermal conductivities along the fast axis (*κ*_f_) and the slow axis (*κ*_s_). One common way to engineer *ρ* in fully dense solids is via nanostructuring^6^, such as fabricating inorganic superlattices^7–11^ or designing symmetry-breaking crystal architectures in a single material^12^. However, such engineered materials have relatively small *ρ* values of less than 20 at room temperature. Conversely, some natural crystalline materials have an intrinsically large *ρ* (for example, graphite^1^ and hexagonal boron nitride (hBN)^13^, with *ρ* ≈ 340 and 90 respectively), but they are often difficult to process scalably for thin film integration. Some of these films may also lack the electrical or optical properties necessary for functional device applications.

To design materials with higher *ρ* that are also suitable for real-world applications, an approach needs to be developed to include three key features: (1) a candidate material with intrinsically high *κ*_f_, usually one with efficient phonon-mediated thermal transport; (2) a method to substantially reduce *κ*_s_ without affecting *κ*_f_; and (3) facile, scalable production and integration of such a material with precise control of the material dimensions (for example, film thickness). Layered van der Waals (vdW) materials such as graphite and transition metal dichalcogenides (TMDs) provide an ideal material platform for designing such high-*ρ* materials. They generally have excellent intrinsic in-plane thermal conductivities (*κ*_||_) in single-crystalline form. Previous studies have also measured record-low thermal conductivities in nanocrystalline vdW films (for example, WSe_2_)^14–17^ and heterostructures^18^. One currently missing capability, however, is an approach for significantly decreasing the out-of-plane thermal conductivity (*κ*_⊥_) while maintaining high *κ*_||_.

## TMD films with interlayer rotations

**Fig. 1:**
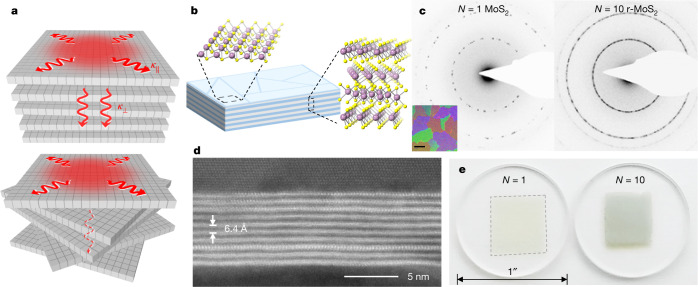
Structure of r-TMD films. **a**, Conceptual strategy for engineering thermal anisotropy in a single material system, using random interlayer rotation in polycrystalline vdW layered materials. **b**, Schematic of an r-MoS_2_ film with random crystalline orientation. **c**, Greyscale-inverted TEM electron diffraction patterns probed from a 500 nm × 500 nm area of a monolayer and an *N* = 10 r-MoS_2_ film. Inset: darkfield TEM image of a monolayer; the scale bar denotes 400 nm and the colours denote different domain orientations from different crystal domains. **d**, HAADF-STEM image of a cross-section of an *N* = 10 r-MoS_2_ film on AlO_*x*_ coated with Al, with an interlayer spacing of 6.4 Å. **e**, Large-area MoS_2_ films transferred onto 1-inch diameter fused silica substrates.

Here we show that such capability is provided by interlayer rotations, as illustrated in Fig. 1a. Interlayer rotation breaks the through-plane translational symmetry at the atomic scale while retaining in-plane long-range crystallinity in each monolayer, thereby providing an effective means for suppressing only *κ*_⊥_. For this, we produce large-area TMD films without interlayer registry (referred to here as r-TMD), which possess long-range crystallinity in-plane and relative lattice rotations at every interlayer interface (Fig. 1b). The films are produced in large-scale using two steps: wafer-scale growth of continuous TMD monolayers (polycrystalline; domain size *D*) and layer-by-layer stacking in vacuum using previously reported methods^19,20^ (see Methods).

**Extended Data Fig. 1:**
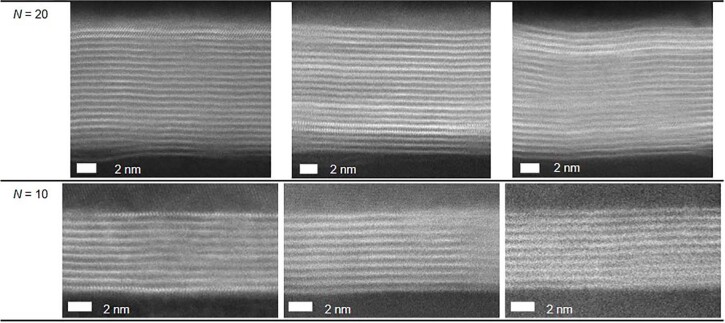
Cross-sectional TEM images of *N* = 20 and 10 r-MoS_2_ on AlO_*x*_. Each set of *N* images are taken from the same sample at different locations. The *N* = 10 films are coated with Al that was electron beam evaporated onto the surface.

**Extended Data Fig. 2:**
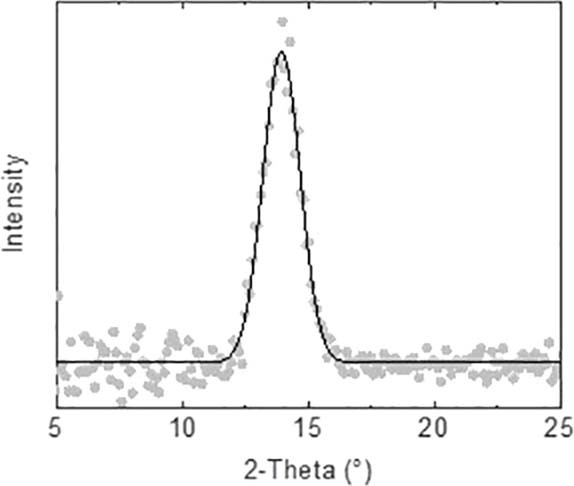
GIWAXS data of *N* = 10 r-MoS_2_. The peak position corresponds to a 2*θ* value of 14˚, which translates to an interlayer spacing of 6.4 Å (scattering direction).

**Extended Data Fig. 3:**
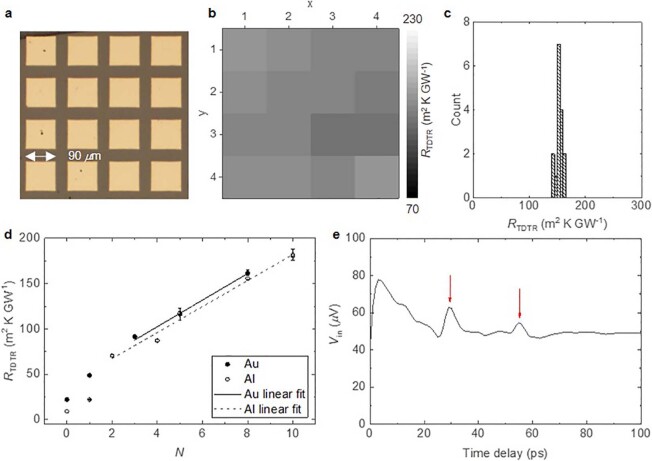
Additional TDTR measurements and details. **a**, Microscope image of an *N* = 10 r-MoS_2_ film coated with a square grid of Al pads. **b**, 4 × 4 TDTR map of *R*_TDTR_ of an *N* = 10 r-MoS_2_ film. **c**, Histogram of *R*_TDTR_ array measurements. **d**, TDTR measurements of *N* ≤ 10 r-TMD films coated with Au or Al. The error bars denote s.d.; number of TDTR measurements per film sample, *n* = 3 for Au samples; *n* = 3–5 for the Al samples. **e**, Picosecond acoustics of a MoS_2_ monolayer on thick sapphire substrate, coated with an Al transducer layer. The *y* axis *V*_in_ is the in-phase signal of the lock-in amplifier. The red arrows indicate the acoustic waves reflected at the Al/MoS_2_ interface.

The transmission electron microscopy (TEM) diffraction (Fig. 1c, left) and darkfield (inset) images from a representative MoS_2_ monolayer show that it comprises large (*D* ≈ 1 μm), randomly oriented crystalline domains, which connect laterally to form a continuous polycrystalline film. The vacuum stacking generates r-TMD films with a precise layer number (*N*) and high-quality interfaces^20^ with interlayer rotation at every stacked interface. The TEM diffraction pattern of *N* = 10 r-MoS_2_ (Fig. 1c, right) shows a ring-like pattern due to the significant increase in the number of diffraction spots, emphasizing the random crystalline orientation in the through-plane direction. Clean and well-defined interfaces can be seen from the cross-sectional high-angle annular darkfield scanning TEM (HAADF-STEM) images of r-MoS_2_ (Fig. 1d and Extended Data Fig. 1; see Methods). The monolayers have a uniform interlayer spacing *d* ≈ 6.4 Å (see Methods and Extended Data Fig. 2), which is close to the expected value (6.5 Å) for twisted MoS_2_ multilayers^21^. Both the growth and stacking steps are scalable, as shown by the optical images of *N* = 1 and *N* = 10 r-MoS_2_ films (~1 cm^2^) in Fig. 1e and as demonstrated later in Fig. 4. The large-scale uniformity of these films also enables precise and reproducible measurements with minimal spatial variation (Extended Data Fig. 3a–c). In our experiments, r-MoS_2_ or r-WS_2_ films with different *N* (up to 22) are transferred onto a sapphire wafer for the measurements of *κ*_⊥_ or suspended over a holey TEM grid (Fig. 3a) for the measurements of *κ*_||_.

## Ultralow out-of-plane conductivity

**Fig. 2:**
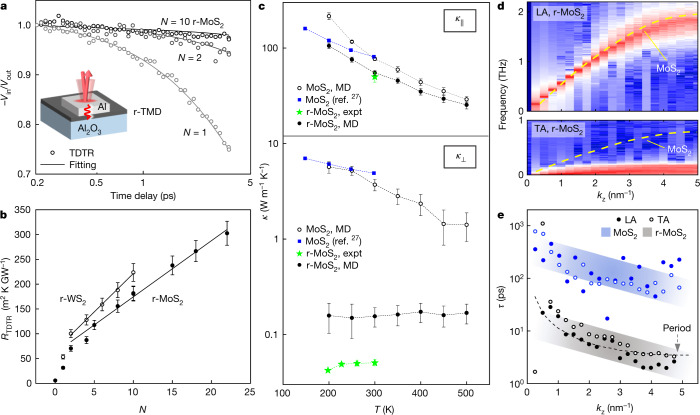
Through-plane thermal properties of r-MoS_2_. **a**, TDTR heat dissipation curves of *N*-layer r-MoS_2_ films. Inset: TDTR sample geometry. **b**, Measured thermal resistances across r-TMD films, where the error bars are the TDTR measurement uncertainties. The thermal conductivities for r-MoS_2_ and r-WS_2_ are calculated from the slope using the formula *R*_TDTR_ = *R*_0_ + *Nd*/*κ*_⊥_, whereby *R*_0_ is the total interfacial thermal resistance. **c**, Experiment and MD simulation results of *κ*(*T*) of MoS_2_ and r-MoS_2_ films. The error bars to the MD simulations originate from the simulation uncertainties. The dotted lines connecting the individual data points are guides to the eye. **d**, LA (top) and TA (bottom) phonon dispersion curves of r-MoS_2_ along the Γ–A direction. The dotted lines denote the acoustic curves corresponding to bulk MoS_2_. **e**, Lifetime of LA and TA phonons parallel to the Γ–A direction in bulk and r-MoS_2_. The dashed line is the LA mode vibration period derived from the dispersion curve in **d**. Source data.

In Fig. 2, we illustrate *κ*_⊥_ of r-TMD films, which is measured using time domain thermoreflectance (TDTR; Fig. 2a, inset; see Methods). A stream of laser pulses (pump) heats up the surface of an Al pad deposited on an r-TMD film on sapphire and produces a temperature-sensitive thermoreflectance signal (−*V*_in_/*V*_out_ in Fig. 2a), which is measured with a probe pulse after a varying time delay (for cooling). Figure 2a shows three representative curves measured from r-MoS_2_ with *N* = 1, 2 and 10. The curves flatten with increasing *N*, suggesting that heat dissipation slows down significantly. Fitting these curves using a heat diffusion model (solid lines, Fig. 2a) enables us to obtain *R*_TDTR_, the total thermal resistance between the Al transducer layer and sapphire across the r-TMD film for different *N*.

Figure 2b shows *R*_TDTR_ versus *N* for r-MoS_2_ (*N* ≤ 22; solid circles) and r-WS_2_ (*N* ≤ 10; open circles) measured under ambient conditions. We make two observations. First, *R*_TDTR_ monotonically increases with *N*. Second, *R*_TDTR_ varies linearly with *N* for *N* ≥ 2. These observations confirm that the through-plane thermal transport in r-TMD films is diffusive in nature, in contrast to the ballistic transport reported in few-layer single-crystalline MoS_2_ (as thick as 240 nm)^22,23^. A single parameter *κ*_⊥_ characterizes the thermal resistance across r-MoS_2_ (or r-WS_2_) using the equation *R*_TDTR_ = *R*_0_ + *Nd*/*κ*_⊥_$,$ where *Nd* is the total film thickness, and *R*_0_ is a constant corresponding to the total interface resistance (r-TMD/Al and r-TMD/sapphire; see Extended Data Table 1). Therefore, we apply linear fitting to the data (*N* ≥ 2) in Fig. 2b (solid lines) to determine *κ*_⊥_ of r-MoS_2_ alone, regardless of the quality and chemical nature of the top and bottom interfaces (see Methods and Extended Data Fig. 3d), which can potentially be altered by metal deposition^24,25^. We measure *κ*_⊥_ = 57 ± 3 mW m^−1^ K^−1^ for r-MoS_2_ and *κ*_⊥_ = 41 ± 3 mW m^−1^ K^−1^ for r*-*WS_2_, which are similar to the lowest value ever observed in a fully dense solid^15^ and comparable to the thermal conductivity of ambient air (~26 mW m^−1^ K^−1^). These values are approximately two orders of magnitude smaller than those of single-crystalline MoS_2_ (2–5 W m^−1^ K^−1^)^26,27^ or WS_2_ (~3 W m^−1^ K^−1^)^27^, despite the r-TMD films having the same chemical composition as their bulk counterparts as well as clean interfaces (Fig. 1d). This strongly suggests that the main difference, the interlayer rotation, is the principal cause for the ultralow *κ*_⊥_ in these r-TMD films. Furthermore, repeating similar TDTR experiments on r-MoS_2_ at different temperatures (*T*) produces a relatively flat *κ*_⊥_(*T*) curve (green stars, Fig. 2c), a behaviour different from the decreasing *κ*_⊥_ with *T* seen in bulk MoS_2_ (blue squares, lower Fig. 2c).

To understand the microscopic mechanisms that give rise to the dramatic reduction in *κ*_⊥_, we carry out homogeneous non-equilibrium molecular dynamics (HNEMD) simulations for the model structures of r-MoS_2_ and bulk MoS_2_ (see Methods and Extended Data Table 2)^28–30^. Figure 2c shows *κ*_||_ and *κ*_⊥_ of r-MoS_2_ (solid circles) and bulk MoS_2_ (empty circles) calculated from our molecular dynamics (MD) simulations at different temperatures. The calculated *κ*_⊥_ drops by a factor of more than 20, from 3.7 ± 0.5 W m^−1^ K^−1^ in bulk MoS_2_ to 0.16 ± 0.04 W m^−1^ K^−1^ in r-MoS_2_ at 300 K, and also does not decrease with *T*, suggesting a transition away from the phonon-limited thermal transport mechanism observed in bulk MoS_2_.

**Extended Data Fig. 4:**
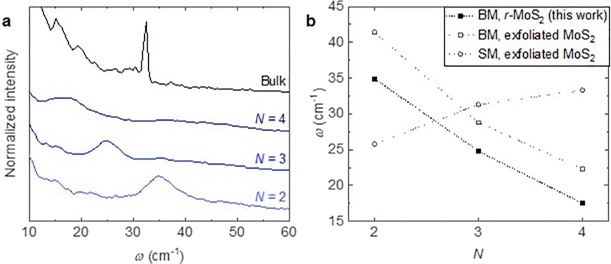
Low-frequency Raman modes of r-MoS_2_. **a**, Raman spectra reflecting the breathing modes (BM) of r-MoS_2_ (blue) and the shear mode (SM) for MoS_2_. **b**, The low-frequency Raman peak positions of r-MoS_2_ and exfoliated MoS_2_. The filled squares indicate the BM peak positions of r-MoS_2_. The open squares indicate the BM peak positions of exfoliated MoS_2_, and the open circles indicate the SM peak positions of exfoliated MoS_2_^72^.

Further analysis of the vibrational spectrum of r-MoS_2_ enables us to break down the reduction in *κ*_⊥_ in terms of the changes in the group velocities (*v*_g_) and lifetimes (*τ*), which are the two factors that determine the thermal conductivity according to Boltzmann transport theory. Figure 2d shows that the *v*_g_ of the through-plane longitudinal acoustic (LA) mode in r-MoS_2_ remains similar to that of bulk MoS_2_ (dashed lines), but the transverse acoustic (TA) modes in r-MoS_2_ undergo extreme softening with their *v*_g_s practically vanishing^31^. This implies a loss of resistance with respect to lateral shear, consistent with the low-frequency Raman spectra of r-MoS_2_ films (see Methods and Extended Data Fig. 4) and previous calculations^32,33^. In addition, the *τ* of both the LA and the TA modes (Fig. 2e) in r-MoS_2_ are more than one order of magnitude smaller than in bulk MoS_2_, with the LA lifetimes being close to the period of the LA mode vibration (dashed line). From these results, the median mean free path $\widetilde{l}$ = *v*_g_*τ* for the LA modes is estimated to be 2 nm, suggesting that the heat-carrying LA modes are strongly scattered and that a larger *D* is unlikely to significantly affect *κ*_⊥_ since *D* >> $\widetilde{l}$. Overall, the strongly suppressed TA modes, indicating a loss of resistance to lateral shear, and the overdamping of the LA modes as the main heat carriers, lead to extremely inefficient thermal transport along the through-plane direction in r-MoS_2_. Along with the nearly temperature-independent *κ*_⊥_, this result suggests a glass-like conduction mechanism.

## In-plane conductivity and anisotropy

**Fig. 3:**
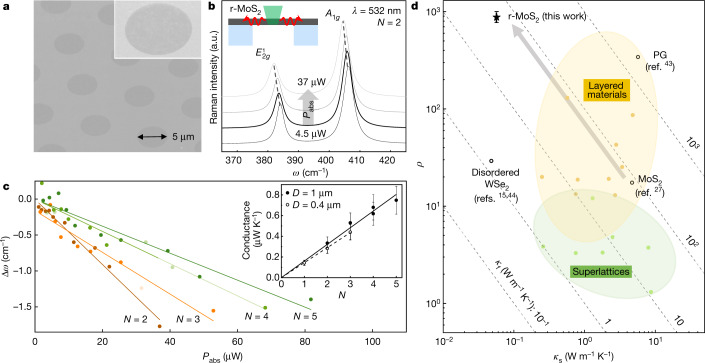
In-plane thermal properties and thermal anisotropy of r-MoS_2_ films. **a**, 45° SEM micrograph of an *N* = 4 r-MoS_2_ film suspended on a TEM grid for Raman thermometry. **b**, Raman spectra of an *N* = 2 r-MoS_2_ film with different absorbed laser powers. Inset: Raman thermometry sample geometry. **c**, A_1*g*_ Raman peak shifts versus power absorbed by r-MoS_2_ films of various *N*. Inset: layer-dependent thermal conductance values (absorbed power divided by temperature increase) in domain size *D* = 1 μm and *D* = 400 nm r-MoS_2_ films. The error bars are the propagated uncertainties from the calculation of the conductance value for each *N*. **d**, Comparison of *ρ* (*y* axis), *κ*_s_ (*x* axis), and *κ*_f_ (diagonal dashed lines) measured for different anisotropic thermal conductors. r-MoS_2_ has an ultrahigh *ρ* close to 900, which is larger than bulk MoS_2_, PG, and disordered layered WSe_2_. The error bar for *ρ* of r-MoS_2_ comes from the propagated uncertainties of the calculated *κ*_⊥_ and *κ*_||_ values. Source data.

In contrast to *κ*_⊥_, *κ*_||_ remains high in our simulations with only a modest reduction compared to the ideal bulk crystal (less than a factor of two at 300 K; Fig. 2c). This is indeed what we observe in our Raman thermometry experiments as discussed in Fig. 3 (see Methods). We direct a focused laser spot (*λ* = 532 nm) at the centre of a suspended r-MoS_2_ film (Fig. 3a; hole diameter of 5 μm, at 15 torr), which increases its temperature (Δ*T*) locally upon absorbing laser power *P*_abs_. Δ*T* is then measured using the temperature-sensitive Raman peak shift (Δ*ω*) using a sensitivity factor (|d*ω*/d*T*|) independently measured for each sample. Examples of Raman spectra measured for *N* = 2 are shown in Fig. 3b.

**Extended Data Fig. 5:**
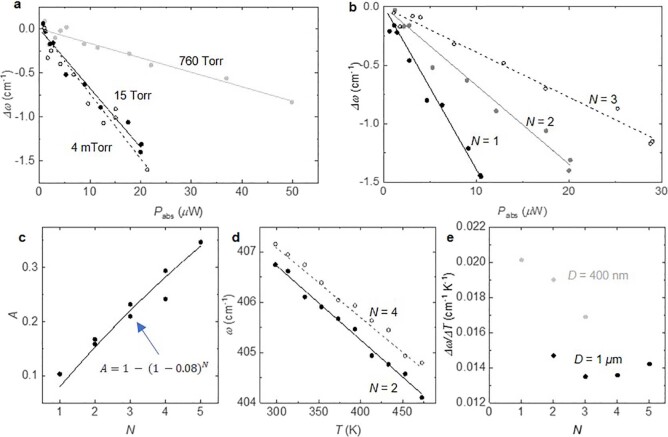
Raman thermometry on r-MoS_2_ films. **a**, Δ*ω–P*_abs_ curves of representative *N* = 2 r-MoS_2_ films at different pressures. The *P*_abs_ values along the *x* axis are normalized to account for the slight differences in beam spot sizes (Δ*r* = 20%). The results at 15 torr and 4 mtorr signify no effect of reducing the pressure to below 15 torr. Δ*ω* was approximately five-fold smaller at atmospheric pressure due to the extra heat loss channel by air. **b**, Δ*ω–P*_abs_ curves of r-MoS_2_ films made up of *D* = 400 nm (grain size) monolayers. **c**, Optical absorption of suspended r-MoS_2_ films, which follows the trend $A=1-{(1-{A}_{0})}^{N}$, where *A*_0_ is the monolayer absorptance. From the fit, *A*_0_ = 0.08 ± 0.003. **d**, *ω–T* calibration measurements of suspended r-MoS_2_ films (*D* = 1 μm), with the *N* = 2 and *N* = 4 data as the representative curves. **e**, *ω–T* slopes versus layer number for all films, with *D* = 400 nm or 1 μm.

**Extended Data Fig. 6:**
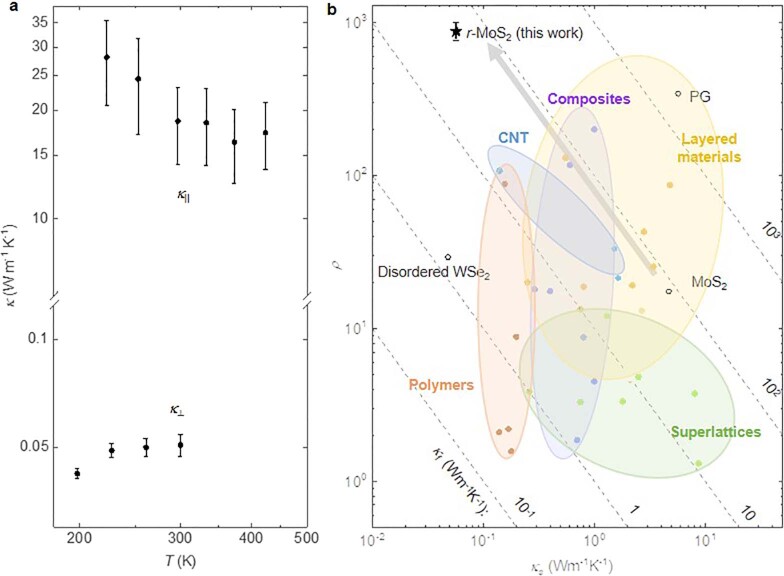
*κ*(*T*) and *ρ* of r-MoS_2_. **a**, *κ*(*T*) of r-MoS_2_, with *κ*_||_ measured using Raman thermometry of *N* = 4 r-MoS_2_, and *κ*_⊥_ measured via TDTR as reported in Fig. 2c. The error bars are the propagated uncertainties from the calculation of the conductance value for each *N*. The error bars denote the propagated uncertainty of the calculations from the input parameters. We observed that *κ*_||_ decreased with *T*, alluding to phonon-mediated heat transport and attesting to the long-range crystallinity of the r-MoS_2_ films in-plane. This was in contrast with the *κ*_⊥_(*T*) behaviour (Fig. 2c), which showed a slightly increasing trend. **b**, Catalogue of experimentally measured anisotropy ratios at room temperature versus slow-axis thermal conductivity (*κ*_s_) of thermally anisotropic materials from literature, by category.

Figure 3c plots Δ*ω* versus *P*_abs_ for r-MoS_2_ with different *N* (2 to 5). The slope of the linear fit (|d(Δ*ω*)/d*P*_abs_|), which is inversely proportional to the in-plane thermal conductance of the film, is plotted in the inset (solid dots; *D* ≈ 1 μm). We again observe a linear relation, which indicates that *κ*_||_ is well defined for r-MoS_2_ independent of *N*, similar to the case of *κ*_⊥_. Using a simple diffusion model with radial symmetry (see Methods and Extended Data Fig. 5c for calculation details and other input measurements), we calculate a high *κ*_||_ value of 50 ± 6 W m^−1^ K^−1^. This value is similar to the predictions of our MD simulations (Fig. 2c) and consistent with previous reports of Raman thermometry on single-crystalline monolayer MoS_2_ (35–84 W m^−1^ K^−1^) at room temperature^34–37^. The *κ*_||_ of these r-MoS_2_ films is close to the intrinsic phonon-limited value despite the films being made of polycrystalline monolayers. This result is further supported by our additional measurements on continuous r-MoS_2_ films with a smaller *D* ≈ 400 nm (open dots, dashed lines, Fig. 3c inset; Extended Data Fig. 5e). The measured value of *κ*_||_ ≈ 44 ± 6 W m^−1^ K^−1^ is within the margin of error of that of the *D* ≈ 1 μm films. This suggests that the phonon mean free path is smaller than 400 nm, which is consistent with previous reports^23,38–42^. Furthermore, the measured in-plane conductance decreases with *T* (Extended Data Fig. 6a). This further confirms the phonon-mediated thermal transport mechanism in-plane, in contrast to the glass-like thermal conduction along the through-plane direction.

Our experiments and calculations confirm that interlayer rotation in r-TMD films results in highly directional thermal conductivity and a direction-dependent thermal conduction mechanism. The rotation significantly reduces *κ*_⊥_ while maintaining high *κ*_||_, leading to an ultrahigh value of *ρ*. We estimate *ρ* ≈ 880 ± 110 at room temperature for the r-MoS_2_ films, higher than that of pyrolytic graphite (PG), which is considered to be one of the most anisotropic thermal conductors (*ρ* ≈ 340)^43^. In Fig. 3d, we compare our result with other previously reported values of *ρ* in phonon-based solids^15,27,43,44^ (for a full comparison, see Extended Data Fig. 6b). Compared to a bulk MoS_2_ crystal (*ρ* ≈ 20)^27^ or disordered layered WSe_2_ (*ρ* ≈ 30)^15,44^, r-MoS_2_ has a significantly larger *ρ* because interlayer rotation reduces only *κ*_⊥_, as denoted by the grey arrow parallel to the equi-*κ*_f_ lines. This also suggests that *ρ* can be made even larger by starting with the monolayers of a layered vdW material with a higher *κ*_||_ value such as graphene.

## Anisotropic vdW heat diffuser

**Fig. 4:**
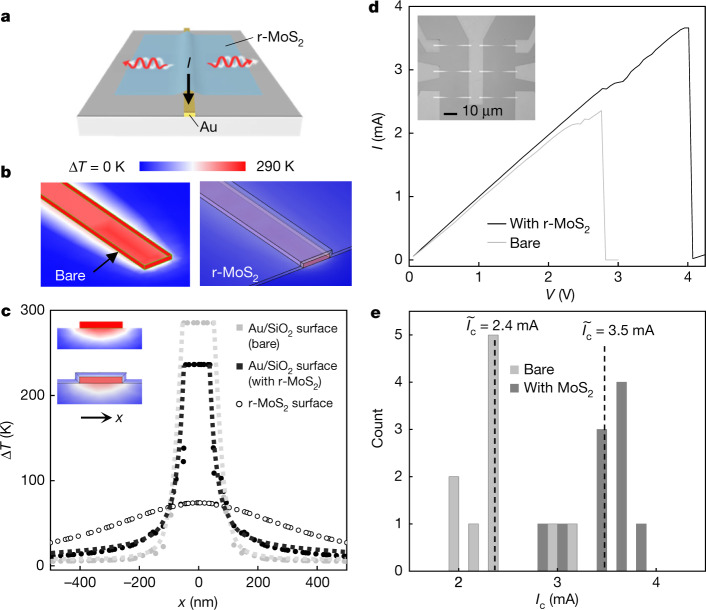
Temperature profiles and heat spreader efficiencies of r-MoS_2_ films on Au electrodes. **a**, Schematic of the sample configuration of r-MoS_2_ draped across a current-carrying Au electrode that is 100 nm wide, 15 nm thick and 10 μm long. **b**, Thermal finite element modelling results of Au electrodes (bare, covered with 10-nm-thick r-MoS_2_) at constant heating power of 8 mW supplied through Joule heating. **c**, Lateral profiles of temperature increases across the Au/SiO_2_ surface (solid dots) and on the r-MoS_2_ top surface (open circles). Insets: cross-sectional temperature distribution of Au electrodes with and without r-MoS_2_, using the same colour scale as in **b**. **d**, *I–V* curve of an Au electrode, with and without *N* = 16 r-MoS_2_. Inset: optical micrograph of six fabricated Au electrodes. **e**, Histogram of *I*_c_ of Au electrodes with and without an *N* = 16 r-MoS_2_ heat spreader and their median values. Source data.

**Extended Data Fig. 7:**
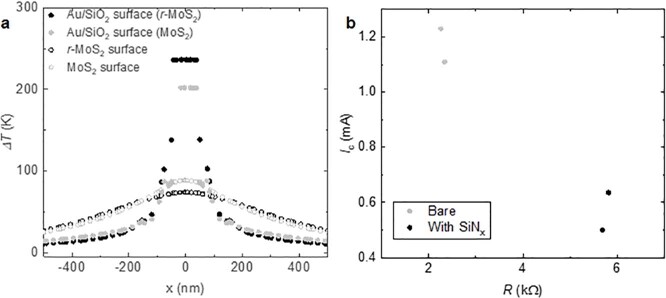
r-MoS_2_ efficacy as a heat spreader. **a**, Finite element simulations of the linear temperature profiles of Au electrodes covered with MoS_2_ and r-MoS_2_. **b**, SiN_*x*_ as heat spreaders for Au electrodes. Electrical properties of 10-nm-thick, 100-nm-wide and 10-μm-long Au electrodes before and after 16 nm SiN_*x*_ film deposition onto the electrodes using plasma-enhanced chemical vapour deposition. In contrast to r-MoS_2_, the direct deposition of an ultrathin inorganic film such as SiN_*x*_ with a comparable *κ* to *κ*_||_ of r-MoS_2_ negatively affects the performance of the Au electrodes.

In Fig. 4, we show that the extreme anisotropy of our r-MoS_2_ films can lead to excellent heat dissipation in-plane from a heat source and drastic thermal insulation in the through-plane direction. Using the COMSOL software, we perform thermal finite-element simulations of a 10-nm-thick r-MoS_2_ film draped over a nanoscale Au electrode (15 nm tall, 100 nm wide) on a 50 nm SiO_2_/Si substrate (Fig. 4a). Our simulation results show that for a fixed power of 8 mW supplied to the Au electrode (near thermal breakdown), the temperature rise ∆*T* of the Au electrode covered by r-MoS_2_ is 50 K lower than that of the bare electrode, thereby demonstrating our film’s effectiveness at spreading heat due to its excellent *κ*_||_ (Fig. 4b, c). Interestingly, the extreme thermal anisotropy of our r-MoS_2_ films provides thermal insulation in the through-plane direction, with much lower MoS_2_ surface ∆*T* values that are only one-third of the value of the bare Au electrode. While single-crystal MoS_2_ displays similar properties, the insulation effect is stronger in r-MoS_2_ (Extended Data Fig. 7a). This implies that heat is efficiently directed away from the hot Au electrode laterally through r-MoS_2_ but not to the surface of r-MoS_2_, making the surface of the entire device significantly cooler.

Our experiments corroborate these simulation results. For this, we fabricate nanoscale Au electrodes with the same geometry and substrate as in our simulation (image shown in Fig. 4d, inset) and transfer *N* = 16 r-MoS_2_ (~10 nm thick) using the vacuum stacking process. Both bare and coated Au electrodes show similar resistance at low currents. At higher currents, current-induced Joule heating leads to the thermally activated electromigration process, which causes the electrodes to fail^45^. Figure 4d compares representative current–voltage (*I–V*) curves measured from a bare and coated Au electrode, which shows that the Au electrode with r-MoS_2_ can carry a larger current without breaking. The histogram of critical current *I*_c_ (maximum current a Au electrode sustains for at least 20 s) measured from 20 electrodes (10 bare and 10 with r-MoS_2_) reveals a ~50% increase in the median *I*_c_ values (Fig. 4e). These results demonstrate our r-MoS_2_ film’s ability to efficiently dissipate Joule heat and keep the electrodes cool, as our simulation predicts. As the electromigration process is dominated by the temperature, the observed increase of *I*_c_ and maximum power before breaking is in good agreement with our simulation in Fig. 4c. Furthermore, we note that the r-MoS_2_ film can be integrated with the Au electrodes using mild conditions that do not affect their electrical properties (Extended Data Fig. 7b).

## Outlook

We expect interlayer rotation to be an effective and generalizable way to reduce *κ*_⊥_ and potentially engineer anisotropic thermal properties in a variety of layered materials. Our results call for a systematic study of the exact relation between *κ*_⊥_ and rotation angle, which could reveal unexpected relationships analogous to the studies of electrical transport in twisted bilayer graphene^46^. Interlayer rotations can be combined with other parameters (such as pressure or interlayer spacing^47,48^) and advanced structures (superlattices and heterostructures^18^) to realize highly tunable *ρ*, allowing for the customization of thermal transport properties with an unprecedented level of directional and spatial control.

## Methods

### Sample preparation

Large-area, polycrystalline transition metal dichalcogenide (TMD) (MoS_2_ and WS_2_) monolayers were grown on SiO_2_/Si substrates in a hot-walled tube furnace via metal-organic chemical vapour deposition adapted from a previously reported protocol^19^. The growth conditions were optimized to produce high-quality monolayer materials with structural characteristics necessary for thermal measurements. These characteristics include large domain size (*D* ≈ 1 μm and 0.4 μm), full monolayer coverage, and laterally stitched grain boundaries.

Briefly, Mo(CO)_6_ and W(CO)_6_ (diluted in N_2_ to 15 torr) were used as the metal precursors for the MoS_2_ and WS_2_ growths, respectively. (C_2_H_5_)_2_S was used as the chalcogen source. All precursors were kept at room temperature. N_2_ and H_2_ were used as carrier gases. Typical growth times were 15–20 h for MoS_2_ at a growth temperature of 525 °C. Typical growth times for WS_2_ were 2 h at a temperature of 650 °C.

To make the r-TMD films, a TMD monolayer was spin-coated with PMMA A8 (poly-methyl methacrylate, 495 K, 4% diluted in anisole) at 2,800 rpm for 60 s, then baked at 180 °C for 3 min. The PMMA-coated monolayer was stacked onto TMD monolayers layer by layer to a target layer number (*N*) and transferred to the desired substrates using a previously reported programmed vacuum stacking method^20^.

#### TDTR samples

The stacked r-TMD films were transferred onto sapphire substrates (Valley Design, C-plane), which were cleaned with Nanostrip solution for 20 min at 60 °C and then rinsed with deionized water. The PMMA layer on the film was removed by immersing the entire substrate in acetone at 60 °C for 1 h. The film was annealed under a 400/100 SCCM Ar/H_2_ environment at 350 °C for 4 h. After cleaning, ~90-nm-thick, 90 μm × 90 μm Al pads were deposited onto the TMD films through a holey TEM grid shadow mask using electron-beam evaporation.

#### Raman thermometry samples

Raman experiments were performed on a different set of films from the TDTR-measured films. First, holey SiN_*x*_ transmission electron microscopy (TEM) grids were cleaned in a N_2_/H_2_ plasma at 100 °C and 180 mtorr for 3 min, followed by the transfer of stacked MoS_2_ films onto the TEM grids. During the transfer process, the PMMA-coated r-MoS_2_ was suspended on holey thermal release tape before contacting the TEM grid. The extra PMMA-MoS_2_ not on the TEM grid was cut away at 180 °C so the PMMA layer was softened. PMMA was removed from r-MoS_2_ on the TEM grid via annealing the film in 400/100 SCCM Ar/H_2_ at 350 °C for 4 h.

### Cross-sectional STEM

The *N* = 10 films were coated with Al that was electron beam evaporated onto the surface, whereas the top surface of *N* = 20 films was bare. The r-MoS_2_ cross-section was prepared using a Thermo Scientific Strata 400 focused ion beam. Protective layers of carbon (~200 nm) and platinum (~1 µm) were deposited on the sample. A cross-section was milled at a 90° angle from the sample using a Ga ion beam at 30 kV. The cross-section was then polished to ~150 nm thickness with the ion beam at 5 kV.

The cross-section was imaged in a Thermo Scientific Titan Themis scanning transmission electron microscope at 120 kV with a probe convergence angle of 21.4 mrad. The *N* = 10 film was imaged at a beam voltage of 120 kV, whereas the *N* = 20 film was imaged at 300 kV. All images were analysed using the open-source software Cornell Spectrum Imager^49^. The high-angle annular dark field (HAADF) image of the sample (see 'TMD films with interlayer rotation' in the main text; Fig. 1d) shows, from top to bottom, the Al crystal lattice along the [110] zone axis, ten layers of MoS_2_ (bright bands), followed by an AlO_*x*_ layer.

### TDTR

We used TDTR to measure the thermal conductivity of our r-TMD films. We used a mode-locked Ti:sapphire laser, which produced a train of pulses at a repetition rate of 74.86 MHz, with wavelength centred at 785 nm and a total power of 18 mW. The steady-state temperature rise at the surface of the samples was <4 K for all temperatures. For the low temperature TDTR measurements, an INSTEC stage was used with liquid nitrogen cooling; the other beam conditions were the same. The laser beam was split into pump and probe beams. A mechanical delay stage was used to delay the arrival of the probe with respect to the pump on the sample surface by changing their optical path difference, before they were focused onto the sample surface through an objective lens. The 1/*e*^2^ radius of the focused laser beams was 10.7 μm. For our measurements, we modulated the pump beam at a frequency of 9.3 MHz so that the thermoreflectance change at the sample surface could be detected by the probe beam through lock-in detection. The ratio of the in-phase and out-of-phase signals from the lock-in was fitted to a thermal diffusion model. The full details of the TDTR measurement can be found elsewhere^50,51^.

#### Calculation of *κ*_⊥_

The modelling required material parameters such as heat capacity (*C*), thickness (*h*), interface conductance (*G*) and thermal conductivity (*κ*) for each layer. Our TDTR samples have three chemically distinct layers with the following structure (from the top): Al/r-TMD/sapphire. In our fitting process, the heat capacities of all materials were adopted from literature^52^. The thickness of Al layer was obtained from picosecond acoustics using a longitudinal speed of sound of 6.42 nm ps^−1^ (Extended Data Fig. 3e). The thickness of the r-TMD film was calculated from the product of *N* and the interlayer spacing (*d*). The latter was measured by performing grazing-incidence wide-angle X-ray spectroscopy (GIWAXS; see GIWAXS section below in the Methods and Extended Data Fig. 2) on the r-TMD films, which gave *d* ≈ 0.64 nm. The total thicknesses of the r-MoS_2_ films were <15 nm; thus, this layer was treated as part of the Al–sapphire interface as a single thermal layer characterized by a single thermal conductance value *G*_1_. We used the bulk value of the volumetric heat capacity of 1.89 J K^−1^ cm^−3^ for the r-MoS_2_ layer. The thermal conductivity of the Al layer was calculated from the Wiedemann–Franz law using the electrical resistance of a transducer layer deposited on a bare sapphire substrate as a reference sample. The thermal conductivity of the sapphire substrate, 35 W m^−1^ K^−1^, was measured using the same reference sample. Thus, the only remaining free parameter to fit for was *G*_1_. To obtain *κ*_TMD_ from *G*_1_, we perform TDTR on various *N*-layer TMD films, then perform a linear fit on the effective thermal resistance (*R*_TDTR_, equal to 1/*G*_1_) versus *N* data points; the slope of the linear fit is inversely proportional to the thermal conductivity, whereas the *y* intercept yields the total interfacial thermal resistances (*R*_0_) of the top and bottom interfaces. In Extended Data Table 1, our *R*_0_ values match the values reported in literature^22,27,53^. We note that, although *R*_0_ changes depending on the chemical nature of the metal–TMD interface, the slope of the *R*_TDTR_–*N* plot (which is used to extract *κ*_⊥_) remains constant, despite the use of different transducer metals, as illustrated in Extended Data Fig. 3d.

For highly anisotropic materials, the anisotropy ratio of an in-plane thermal conductivity to a through-plane conductivity should be included in the thermal model. Despite the ultrahigh thermal anisotropy expected of our r-TMD films, our through-plane thermal conductivity measurements were probably not sensitive to the thermal conductivity anisotropy given the thinness of our r-TMD films. Hence, we assumed a one-dimensional thermal transport model and neglected the in-plane thermal transport in our calculations. We found that the effect of the anisotropy was significant only at a smaller modulation frequency (*f* = 1.12 MHz) and 1/*e*^2^ beam radius of ~3.2 μm, and so we deliberately chose a larger *f* and a 1/*e*^2^ beam radius to reduce the sensitivity of our TDTR signal to the in-plane thermal transport.

### Raman thermometry

We followed a similar procedure from previous reports^34,54,55^ with the modification of lower pressures during measurement. All the Raman measurements were performed using a Horiba Raman spectrometer with a laser excitation wavelength of 532 nm and a long-working distance, 50× objective lens (numerical aperture (NA) = 0.5). The r-MoS_2_ A_1*g*_ peak shift (*ω*) versus temperature (*T*) relation was calibrated using a temperature-controlled, low-vacuum-compatible Linkam stage. For all our Raman measurements, we used the A_1*g*_ peak since this out-of-plane vibrational mode is less sensitive to in-plane strain^56^. The *ω*–*T* calibration measurements were performed at atmospheric pressure and with low laser powers. The stage was purged with dry N_2_ gas throughout the calibration step to prevent oxidative damage to the film at high temperatures. Extended Data Fig. 5d shows representative *ω*–*T* calibration curves for *N* = 2 and *N* = 4 r-MoS_2_ films, where a linear fit was performed to obtain the temperature-dependent Raman coefficients. This process was repeated for r-MoS_2_ films with different domain sizes *D* (400 nm and 1 μm) for *N* = 1–3 (Extended Data Fig. 5e).

To measure the in-plane thermal conductivity (*κ*_||_) of our films, the laser power (*P*) was varied and the corresponding Δ*ω* values were recorded. The in-plane thermal conductance was obtained from the reciprocal of the slope of the Δ*ω*–*P* linear fit, which is illustrated for r-MoS_2_ films with *N* = 2–5 and *D* = 1 μm in Fig. 3c and for r-MoS_2_ films with *N* = 1–3 and *D* = 400 nm in Extended Data Fig. 5b. As thermal conductivity changes with temperature, laser powers were kept below 250 μW to induce a relatively small Δ*T* in the film and ensure that the value of *κ*_||_ remained relatively constant. This was verified from the observation of a linear Δ*ω*–*P* regime for *P* < 250 μW. Any higher laser powers caused the Δ*ω–P* curve to deviate from the linear regime with $\frac{{{\rm{d}}}^{2}\omega }{{\rm{d}}{P}^{2}} < 0$. This indicates that the local film temperature increased faster at higher *P* > 250 μW, which signified that the thermal conductivity could no longer be assumed to be constant. Instead, the thermal conductivity decreased with increasing temperature, consistent with the *T*-dependent Raman measurements.

The Δ*ω*–*P* measurements were conducted at a pressure of 15 torr to eliminate any heat loss to air. We verified that a lower pressure down to 4 mtorr gave rise to similar Δ*ω* values as the measurements at 15 torr (Extended Data Fig. 5a), weighted by the beam spot size.

The other relevant input quantities for our thermal calculations were obtained as follows: the beam spot radius (*r*_0_) was estimated using the knife-edge method, whereby a one-dimensional Raman map was taken across a gold step edge on an Au-patterned silicon chip, and the spatial distribution of the integrated peak intensities was fitted to an error function. We measured *r*_0_ = 0.71 ± 0.09 μm. The laser powers were measured using a Thorlabs standard silicon photodiode power sensor. The r-MoS_2_ absorbance $A=\frac{{\rm{Absorbed\; light\; intensity}}}{{\rm{Incident\; light\; intensity}}}$ was measured at room temperature on a white-light microscope with a 532 nm band-pass filter and a low-NA condenser aperture. We measured the light intensity transmitted through and reflected from a r-MoS_2_ film suspended on a TEM grid, then compared it against a blank TEM grid. The data were collected using a 12-bit SensiCam QE CCD camera. The pixel intensities were analysed using ImageJ. The values for *A* were calculated using the formula *A* = 1 − *T* − *R*. We measured *A*(*N*) for *N* = 1–5, then fitted *A* to a power law. *A*(*N*) was found to follow the relation $A=1-{0.92}^{N}$ (Extended Data Fig. 5c), which matched previous reports^20^. We use the value *A* measured at room temperature for our Raman analysis.

#### Calculation of *κ*_||_

To obtain the value of *κ*_||_, we used the two-dimensional thermal diffusion equation with a radial symmetry, following previous reports of Raman thermometry of two-dimensional films^54,55^. We assumed a Gaussian laser profile $q(r)=\frac{{PA}}{({\rm{\pi }}{{r}_{0}}^{2})t}\,{\rm{\exp }}\left(-\frac{{r}^{2}}{{{r}_{0}}^{2}}\right)$. We solved for *κ*_||_ numerically using the following equations:

$${\kappa }_{{\rm{||}}}\frac{1}{r}\frac{{\rm{d}}}{{\rm{d}}r}\left(r\frac{{\rm{d}}{T}_{{\rm{susp}}}(r)}{{\rm{d}}r}\right)+q(r)=0{\rm{;}}r < R$$

$${\kappa }_{{\rm{||}}}\frac{1}{r}\frac{{\rm{d}}}{{\rm{d}}r}\left(r\frac{{\rm{d}}{T}_{{\rm{supp}}}(r)}{{\rm{d}}r}\right)-\frac{G}{t}[{T}_{{\rm{supp}}}(r)-{T}_{{\rm{a}}}]=0{\rm{;}}r > R$$

applying the boundary conditions

$${T}_{{\rm{susp}}}(R)={T}_{{\rm{supp}}}(R)$$

$$\frac{{\rm{d}}{T}_{{\rm{susp}}}}{{\rm{d}}r}(R)=\frac{{\rm{d}}{T}_{{\rm{supp}}}}{{\rm{d}}r}(R)$$

where $r$ is the distance from TEM hole centre, $P$ is the laser power, $t$ is the film thickness, $R$ is the TEM hole radius, $T$ is the film temperature where ${T}_{{\rm{susp}}},{r}\le R$ and ${T}_{{\rm{supp}}},{r}\ge R$, ${T}_{{\rm{a}}}$ is the ambient temperature, $A$ is the fraction of laser power absorbed, and *G* = 10 MW m^−2^ K^−1^ is the interfacial thermal conductance between r-MoS_2_ and SiN_*x*_.

We solved for the expression of *T*(*r*) and obtained an expression for the average temperature measured by the Raman shift

$${T}_{{\rm{m}}}=\frac{{\int }_{0}^{R}T(r)r\,\times \,\exp (-\frac{{r}^{2}}{{{r}_{0}}^{2}}){\rm{d}}r}{{\int }_{0}^{R}r\,\times \,\exp (-\frac{{r}^{2}}{{{r}_{0}}^{2}}){\rm{d}}r}$$

*κ*_||_ was obtained by substituting the experimentally measured value for ${T}_{{\rm{m}}}$ and solving the above equation numerically for *κ*_||_. We calculated *κ*_||_ for each *N*, and we reported the average value in the main text.

The total measurement uncertainty reported in the main text was calculated based on the error assessment for individual parameters. We used an approximate analytical solution

$${\triangle T=T}_{{\rm{m}}}-{T}_{{\rm{a}}}\approx -\left(\frac{{P}_{{\rm{abs}}}}{{\kappa }_{{||}}}\right)\left(\frac{1}{2{\rm{\pi }}{rt}}\right){\rm{ln}}\left(\frac{R}{{r}_{0}}\right)$$

$${\kappa }_{{\rm{||}}}\approx \left({\left(\frac{\triangle \omega }{\triangle P}\right)}^{-1}\left(\frac{\triangle \omega }{\triangle T}\right)\right)\left(\frac{{A}_{0}}{2{\rm{\pi }}d}\right){\rm{ln}}\left(\frac{R}{{r}_{0}}\right)$$

where $\omega$ is the Raman frequency of A_1*g*_ peak, ${A}_{0}$ is the absorption of the monolayer, and $d$ is the thickness of a monolayer. The difference between the full numerical solution and this analytical form is below 3%. We identified the following independent quantities that carry uncertainty for consideration in our overall uncertainty estimation of *κ*_||_.

1. ${\frac{\triangle P}{\triangle T}=(\frac{\triangle \omega }{\triangle P})}^{-1}\left(\frac{\triangle \omega }{\triangle T}\right)$: the associated uncertainty was derived from the error in the linear fit of $(\frac{\triangle \omega }{\triangle P})$ and $\left(\frac{\triangle \omega }{\triangle T}\right)$ for every sample measured. The total uncertainty in the average $\frac{\triangle P}{\triangle T}$ value was 9% for *D* = 1 μm and 8% for *D* = 400 nm.
2. $\left(\frac{{A}_{0}}{2{\rm{\pi }}{d}_{0}}\right)$: the uncertainty in *A*_0_ from the *A*(*N*) fit was 4%.
3. ${\rm{ln}}\left(\frac{R}{{r}_{0}}\right)$: the uncertainty originated from the uncertainty in *r*_0_. From 14 repeated measurements of the beam spot size using the knife-edge method, we calculated the standard deviation of *r*_0_ to be 12%, which translated to an uncertainty in the expression ${\rm{ln}}\left(\frac{R}{{r}_{0}}\right)$ to be 9%.

Total uncertainty in *κ*_||_: 13% (for both *D* = 1 μm and *D* = 400 nm).

#### Variable pressure Raman thermometry measurements

Previous Raman thermometry measurements on graphene films^57^ and carbon nanotubes^58^ had shown an appreciable difference between measurements performed in air and at lower pressures, as well as in different gaseous environments. We extended the same precaution and repeated our Raman thermometry measurements at low pressures to reduce heat dissipation to air, an extra heat loss channel that would lead to an overestimation of the thermal conductivity of the r-MoS_2_ films.

Our Δ*ω*–*P* measurements in Fig. 3 were conducted at a pressure of 15 torr. In Extended Data Fig. 5a, we compared the Δ*ω* values of *N* = 2 r-MoS_2_ at three different *P* (1 atm, 15 torr and 4 mtorr), after correcting for the different laser spot sizes.

#### Temperature-dependent Raman thermometry for *κ*_||_

We performed Raman thermometry while varying the ambient temperature *T*_a_ using a Linkam stage. No oxidation or sample damage was detected for any of the temperatures used. We performed the same Δ*ω–P* measurements and calculated the *κ*_||_ value of for each *T*_a_. We plot a *κ*_||_ versus *T* curve, where the *x* axis is *T* = *T*_a_ (Extended Data Fig. 6a).

We note that the measured values of *κ*_||_ here were lower than the room temperature values reported in the main text. We ascribe this to the sub-optimal growth conditions for the constituent monolayers used for this sample.

### r-MoS_2_ heat spreader experiments (electromigration of Au nanoelectrodes)

All Au electrodes were fabricated on Si substrates with 50 nm dry SiO_2_ in three nanopatterning and deposition layers: (A) the nanoelectrodes (10 μm long, 100 nm wide, 15 nm thick); (B) the contact pads that would interface with the external electronics (200 μm long, 300 μm wide, 100 nm thick); and (C) the leads connecting the nanoelectrodes and the contact pads (~1,000 μm long, 50 μm wide, 15 nm thick).

We first defined the leads (B) and then the contact pads (C) using standard photolithography, and electron-beam evaporation of Ti (1 nm)/Au and lift-off. The final step was defining the nanoelectrodes (A) using electron-beam lithography, deposition of 15 nm Au, and lift-off.

#### Electron-beam lithography

We used a bilayer of resists: copolymer P(MMA-MAA 11%) in ethyl lactate and 950 K PMMA A4. The writing was executed with a Raith EBPG 5000 Plus E-beam writer with the beam conditions of 25 nA current, dose of 1,200 μC cm^−2^, 300 μm aperture size, 100 kV accelerating voltage.

#### Film transfer

After the nanoelectrodes, leads and pads were fabricated, the device was cleaned with an O_2_ plasma for 30 s to remove any resist residue and to promote adhesion of the r-MoS_2_ film to the Au electrodes and the SiO_2_ surface. A PMMA coated *N* = 16 r-MoS_2_ film was transferred onto the electrodes using the same process as the stacking method as outlined above. The PMMA on the r-MoS_2_ film was removed by immersing the entire chip in toluene at 60 °C for 1 h.

#### Electrical measurements

All measurements were performed in ambient conditions with a home-built probe station in a two-probe geometry. To measure *I*_c_ in Fig. 4d, we swept the voltage bias in only one direction at a rate such that the rate in current increase is 0.05 mA per 20 s.

For comparison, we deposited SiN_*x*_ onto Au electrodes (10 μm long, 10 nm thick, 100 nm wide) using plasma-enhanced chemical vapour deposition with the following conditions: 10 s deposition at 90 °C and 10 torr and 1,000 W plasma power, with 25 SCCM/35 SCCM SiH_4_ and N_2_ as the precursors. The film thickness was measured via ellipsometry to be 16 nm.

### Computational methodology

#### Structural models

Structural models were created according to an algorithm previously described in literature^59^, which was implemented in Python using the atomic simulation environment package^60^. The structure models were subsequently relaxed using an analytic bond-order potential^61^ and implemented in the LAMMPS package^62^.

The main r-MoS_2_ model used in the simulations described here comprised 10 randomly stacked layers with a total of 10,152 atoms. The 10 layers came in pairs; each pair was related by a 60° rotation. The four primitive angles present in the stack are 16.1, 25.28, 34.72 and 43.9°. Due to strain, each layer contained a different number of atoms in accordance with strains of around 10%.

The bulk structure used in the MD simulations comprised 40 layers (20 conventional unit-cells) with a total of 26,880 atoms and cell vectors of 44.44, 43.98 and 243.57 Å.

#### HNEMD simulations

**Extended Data Fig. 8:**
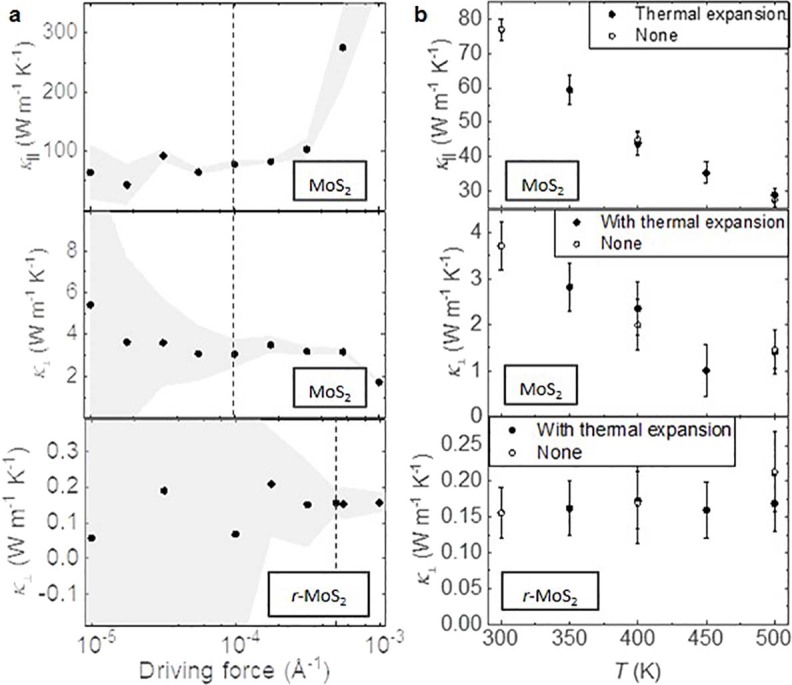
Optimization of the MD simulations for *κ* calculations. **a**, Optimization of the driving force of the system, where the grey zone denotes the error. **b**, Effect of thermal expansion on *κ*.

The interatomic potential in our simulations produces the expected slight increase in the interlayer spacing in r-MoS_2_ and yields thermal conductivities and phonon dispersions of bulk MoS_2_ that agree with previous experimental observations and Boltzmann transport calculations based on density functional theory^38^, confirming our MD model’s suitability for this study. The structures described above were driven by an optimized driving force (Extended Data Fig. 8a) and subsequently relaxed, after which the thermal conductivity was computed using HNEMD simulations^63^ and implemented in the graphics processing units molecular dynamics (GPUMD) package^29^. We also included the effects of thermal expansion in the simulations (Extended Data Fig. 8b). The calculated *κ*_⟂_ values of r-MoS_2_ are higher than the experimental values. We attribute the discrepancy to our neglecting any quantum effects and all boundary scattering in our simulations. Including such effects could further improve results from simulations. Statistics and averages were gathered from ten independent simulation runs for each system and temperature. The other parameters used in these simulations are compiled in Extended Data Table 2.

#### Phonon dispersion and lifetimes

We first generated the bulk MoS_2_ phonon dispersion in the harmonic limit as a reference to phonon dispersion calculations using MD simulations. We computed the harmonic (0 K) phonon dispersion using the PHONOPY package^64^. Forces were computed for 6 × 6 × 2 supercells using the LAMMPS code. Lifetimes were calculated using the lowest applicable order of perturbation theory using the PHONO3PY package^65^, which also provided us with the thermal conductivity as obtained from a direct solution of the Boltzmann transport equation^66^. In these calculations, the Brillouin zone was sampled using a 10 × 10 × 10 Γ-centred q-point mesh, which was chosen for consistency with the supercell used in the HNEMD simulations.

Next, we compared the dispersion of bulk MoS_2_ to that calculated using MD simulations to verify the accuracy of our MD simulations for calculating the phonon dispersion of r-MoS_2_. For both bulk and r-MoS_2_, we extracted the phonon dispersions and lifetimes at 300 K by analysing the longitudinal and transverse current correlation functions generated by MD simulations in the microcanonical (NVE) ensemble using dynasor^67^. The MD simulations details were otherwise identical to the HNEMD simulations. The obtained correlation functions were Fourier transformed and fitted to peak shape functions corresponding to (over)damped harmonic oscillators using the full expressions given in the dynasor paper^67^ to obtain phonon frequencies and lifetimes.

#### Finite-element analysis

We used the COMSOL software to simulate the steady-state temperature distribution in a Au electrode on a SiO_2_/Si substrate. Our geometry contains an Au electrode that is 100 nm wide, 15 nm thick, and 10 μm long on a 50-nm-thick SiO_2_ layer on a Si substrate. We layer a 10-nm-thick MoS_2_ film onto the Au electrode and the SiO_2_ layer. For the thermal anisotropy consideration, we define the thermal conductivity slow axis direction to always be perpendicular to the film’s bottom surface in contact with the substrate or the Au electrode, including the Au electrode side walls.

We supply the Au electrode with 8 mW uniformly over the entire volume as the heat source, matching the power conditions at which the Au electrode fails in our experiments. As the boundary condition, we set the bottom surface on the Si substrate to be at 293.15 K. We also account for all the interfacial thermal resistances between heterogeneous surfaces in our calculations, which include r-MoS_2_/Au, r-MoS_2_/SiO_2_, Au/SiO_2_, SiO_2_/Si (refs. ^68–71^). All effects of radiation are neglected as they do not affect the temperature values in our simulations.

### Low-frequency Raman measurements

The low-frequency Raman spectra of *N* = 2, 3 and 4 r-MoS_2_ films, along with the spectrum for MoS_2_, are shown in Extended Data Fig. 4a. From the layer-dependence of the peak positions, we assigned these to be the breathing modes of MoS_2_ (ref. ^72^). We did not observe any peaks corresponding to shear modes in r-MoS_2_. Our findings agree with theoretical studies of low-frequency Raman modes of twisted MoS_2_ bilayers, which showed that the shear mode peaks redshift to below the detection capabilities (2 cm^−1^)^73^. The positions of the breathing mode peaks of the r-MoS_2_ films were close to those in exfoliated few layer MoS_2_ (Extended Data Fig. 4b) as reported in literature^72,74^. This observation agreed with our MD simulations that suggested that the transverse vibrational mode was suppressed by interlayer rotation, while the longitudinal vibrational mode was retained.

### GIWAXS

The GIWAXS measurement was performed using SAXSLAB (XENOCS)’s GANESHA (lab-source Cu Kα, photon flux ~108 photons s^−1^) to characterize the interlayer spacing of r-MoS_2_ films. An *N* = 10 r-MoS_2_ film was prepared on a SiO_2_/Si substrate. The incidence angle of the X-ray beam was 0.2° and the integration time was ~60 s. Radially integrating the two-dimensional diffraction images along the out-of-plane direction produced the diffraction spectrum along the *c* axis shown in Extended Data Fig. 2.

The peak position of 14° corresponded to an interlayer spacing of 6.4 Å in the [0 0 1] direction, which matched previous reports of r-TMD films^20^.

## Online content

Any methods, additional references, Nature Research reporting summaries, source data, extended data, supplementary information, acknowledgements, peer review information; details of author contributions and competing interests; and statements of data and code availability are available at 10.1038/s41586-021-03867-8.

### Source data

Source Data Fig. 2

Source Data Fig. 3

Source Data Fig. 4

## Figures and Tables

**Extended Data Table 1:**
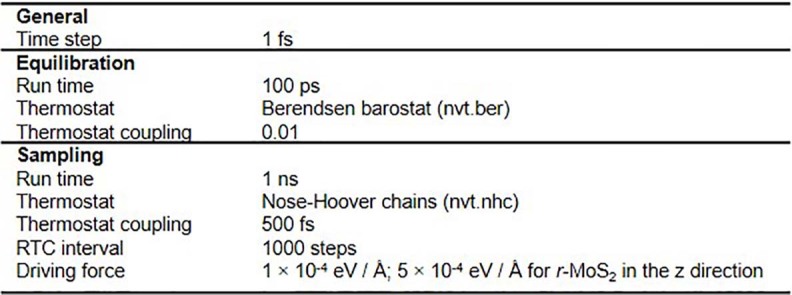
Comparison of literature thermal boundary resistances with experimentally measured values

**Extended Data Table 2:**
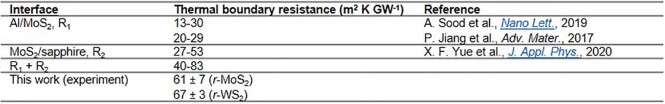
Parameters used in the calculations of the thermal conductivity using GPUMD

## Data Availability

The data that support the findings of this study are available from the corresponding authors on reasonable request. Source data are provided with this paper.

## Code availability

All code used in this work is available from the corresponding authors on reasonable request.
